# Analysis of North American Rheumatoid Arthritis Consortium data using a penalized logistic regression approach

**DOI:** 10.1186/1753-6561-3-s7-s61

**Published:** 2009-12-15

**Authors:** Pascal Croiseau, Heather J Cordell

**Affiliations:** 1 Institute of Human Genetics, Newcastle University, International Centre for Life, Central Parkway, Newcastle upon Tyne NE1 3BZ UK

## Abstract

We applied a penalized regression approach to single-nucleotide polymorphisms in regions on chromosomes 1, 6, and 9 of the North American Rheumatoid Arthritis Consortium data. Results were compared with a standard single-locus association test. Overall, the penalized regression approach did not appear to offer any advantage with respect to either detection or localization of disease-associated polymorphisms, compared with the single-locus approach.

## Background

Penalized regression approaches are an attractive option for the analysis of large numbers of predictor variables (such as genotypes at many genetic loci) that may influence a response variable (such as disease status). Most genome-wide studies use single-locus association tests such as the Cochran-Armitage trend test, or, equivalently, logistic regression with a single predictor variable (encoding the effect of a particular locus) included in the regression equation at any given time. Theoretically, regression methods allow the simultaneous inclusion of several different variables in the regression equation, e.g., variables coding for genotype rather than allele effects (thus modeling "dominance"), or variables that encode effects at several different loci. However, standard regression methods fail when the sample size (the number of people) is small compared to the number of predictors.

Standard linear regression can be formulated as finding the vector *β *of parameter estimates (regression coefficients) *β*_*j *_(*j *= 1,...,*p*) at *p *predictors that minimizes the sum of squared differences ,

where, for person *i*, *y*_*i *_is a quantitative outcome variable and *x*_*ij *_is a predictor variable (such as a genotype variable taking values 0, 1, or 2 according to the number of risk alleles at locus *j*). In penalized regression, one minimizes this function subject to a constraint on the coefficients such as  or as . The theory of Lagrange multipliers suggests that this problem may be re-formulated as minimizing

where *g*(*β*_0_, *β*)corresponds to the original sum of squared differences, *h*(*λ*, *β*)is a penalty term, and *λ *is a tuning parameter (or vector of parameters) that controls the strength of penalization. Ridge regression [1] uses a so-called L_2 _penalty

producing coefficients that are scaled down or "shrunk" towards zero and prediction models that often perform better that least-squares owing to a bias-variance trade-off [2]. All predictors remain in the model, some with small coefficients. The lasso [3],

uses an L_1 _penalty, resulting in both shrinkage and variable selection, in that many of the coefficients become set to zero. Zou and Hastie [2] proposed a penalty *h*(*λ*_1_, *λ*_2_, *β*)that is a convex combination of the lasso and ridge penalties

which they termed the naïve elastic net. However, this method can over-shrink the coefficients and performs poorly unless either *λ*_1 _or *λ*_2 _is close to 0. Zou and Hastie [2] therefore instead proposed using a modified version of the elastic net that essentially scales up the naïve elastic net coefficients by a factor of (1 + *λ*_2_).

The naïve and modified elastic net approaches enjoy a grouping property whereby predictors that are highly correlated tend to have similar coefficient estimates [2]. An alternative penalization method that enjoys a similar property is the group lasso [4], which minimizes the objective function *f*(*β*_0_, *β*) = *g*(*β*_0_, *β*) + *h*(*λ*, *β*) with

(i.e., half the sum of squared differences) and penalty term

Here, the predictors are divided into *G *groups (*g *= 1,...,*G*) and *f*_*g *_and *l*_*g*_indicate the first and last predictor in group *g*. The penalty term in the group lasso is intermediate between the L_1 _penalty of the lasso and the L_2 _penalty used in ridge regression and, as pointed out by Wu and Lange [5], provides a natural coupling between parameters in the same group. Wu and Lange [5] actually propose an alternative approach, which is to minimize the objective function *f*(*β*_0_, *β*) = *g*(*β*_0_, *β*) + *h*(*λ*, *β*), with *g*(*β*_0_, *β*) equal to either half the sum of squared differences as above (denoted *l*_2 _regression) or to  (denoted *l*_1 _regression), with the penalty term taking the form

This is similar in form to the naïve elastic net penalty, except that, like the group lasso, it uses ||*β*_*g*_||^2 ^instead of  in the group-specific penalty controlled by *λ*_2_.

Penalization is an attractive option in genetic studies because it allows the grouping of predictors that relate to the same genetic variant or region, and also because we genuinely expect the vast majority of loci to have regression coefficient 0. Although originally developed for quantitative outcomes, penalization methods have been extended to deal with binary outcomes (such as disease). Penalization is achieved by minimizing an objective function *f*(*β*_0_, *β*) = *g*(*β*_0_, *β*) + *h*(*λ*, *β*) with the penalization term *h*(*λ*, *β*) taking one of the forms above, and *g*(*β*_0_, *β*)equalling minus one [6] or two [7] times the log likelihood of the data. Software implementations include the R package "glmnet", which fits the lasso or elastic-net regularization path for linear, logistic, and multinomial regression models, and the R package "grplasso," which fits a variant of the group lasso approach for binary outcome data.

## Methods

### Data

We analyzed the North American Rheumatoid Arthritis Consortium (NARAC) data, consisting of 868 rheumatoid arthritis (RA) cases and 1194 controls genotyped at 545,080 single-nucleotide polymorphisms (SNPs) across 22 autosomal chromosomes. These data were recently used in combination with additional samples [8] to perform genome-wide association analysis, confirming previously proposed associations between disease and variants in *HLA *and *PTPN22*, and also reporting a new locus on chromosome 9. We therefore focused on these regions for application of our penalized regression approach.

### Quality control

We used the software PLINK [9] to perform basic quality control checks. SNPs were excluded based on a SNP genotype call rate of <95%, minor allele frequency <1%, and Hardy-Weinberg equilibrium (HWE) *p*-value < 10^-7^. We also removed individuals with >5% missing genotypes. We used multidimensional scaling of the Genetic Analysis Workshop (GAW) 16 data, together with publicly available HapMap data on 210 unrelated individuals from four populations, to confirm that the individuals from the GAW data had European ancestry and were not related.

### Single-locus analysis

We used PLINK to perform a Cochran-Armitage trend test at each SNP. Unlike Plenge et al. [8], we made no attempt to correct for population stratification, as we wished to compare our single-locus results with those from group lasso penalized regression, which does not (in its current software implementation) allow inclusion of additional covariates such as principal-component scores from an eigenvector analysis [10].

### Penalized regression analysis using the group lasso procedure

We applied the group lasso procedure proposed by Meier et al. [6] implemented in the R package "grplasso" to SNP data in the three regions of association (chromosomes 1, 6, and 9) detected by Plenge et al. [8]. Because the software required data to be available at all predictor variables, PLINK was first used to impute any missing genotypes on the basis of linkage disequilibrium (LD) patterns with observed genotypes. We chose this particular penalization approach and software because it is one of the few available methods that deal with binary (case/control) as opposed to quantitative outcomes, and because we were attracted by the natural coupling of parameters that could potentially be achieved through use of the group lasso penalty term.

Consideration of groups of predictors simultaneously could be useful if one wished to include more than one predictor per SNP (e.g., to model genotype effects rather than allelic effects, or interactions only in the presence of main effects) or to impose some other grouping based on (for example) biological function. However, in our analyses, we used only a single predictor variable per locus (coded 0, 1, or 2 according to the number of variant alleles), and thus each SNP formed a group by itself.

The group lasso estimator [6] is defined as the minimizer *β *of the convex function

where *l*(*β*_0_, *β*) is the logistic regression log-likelihood function and the function  is used to rescale the penalty with respect to dimensionality of the parameter vector for group *g *(not relevant here). The choice of the tuning parameter *λ *controls the amount of penalization. A natural way to estimate *λ *is to use cross-validation [5], however this can be very time consuming, particularly when coupled with the bootstrapping approach that we describe below. Instead we used the simpler proposal by Meier et al. [6] to take *λ *equal to log(*G*), where *G *is the number of groups, in our case the number of SNPs to be fitted in the model. Thus, *λ *varied from log(1000) = 6.9 to log(7000) = 8.85 in the results described below.

The output from a penalized regression procedure consists of an estimated regression coefficient for each predictor in the model: model selection is performed by estimation rather than hypothesis testing [5]. Because we do not have any measure of the variability of the estimated coefficient, interpretation of the importance or significance of any particular predictor can be problematic. Ideally, we would like to present results in the form of a significance test for each coefficient in order to perform comparisons with standard single-locus tests of association. To address this limitation, we used a bootstrap: the penalized regression procedure was performed 50 times on 50 different bootstrap replicates constructed by selecting observations (people) with replacement from the original sample. This allowed us to estimate the variance of each regression coefficient. We then constructed a *z*-score at each locus by dividing the observed regression coefficient by its estimated standard error, and converted this to a *p*-value, assuming the *z*-score to be normally distributed. This procedure is not, strictly speaking, correct, because penalized regression does not enjoy the asymptotic properties of standard regression procedures: shrinkage of the regression coefficients means their distribution cannot be assumed to be asymptotically normal. However, we hoped that this procedure would provide us with a ballpark estimate of the relative significance of the regression coefficients (relative to one another), even if the exact significance levels could not be considered reliable.

## Results

**Figure 1:**
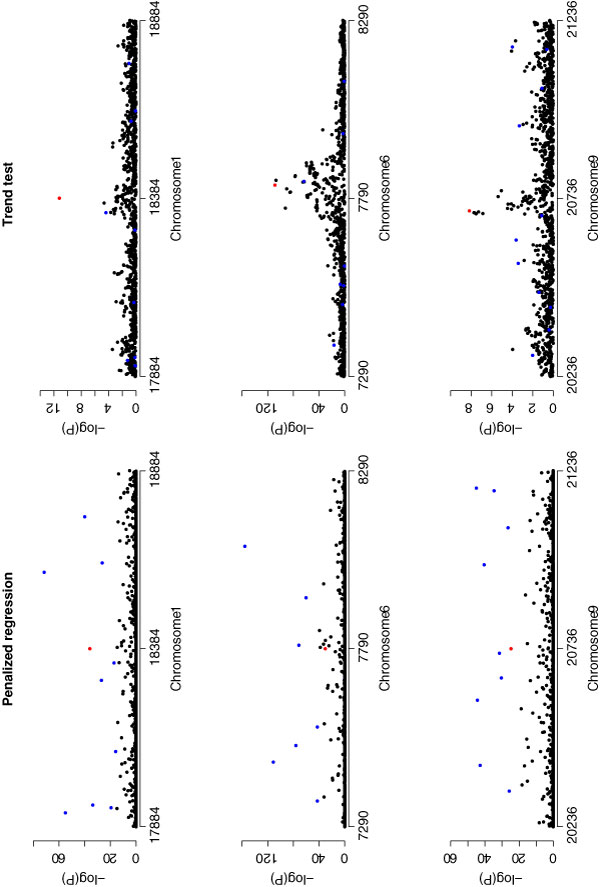
**Results from bootstrap penalized regression and single-locus logistic regression (trend test) analysis**. Results are shown in terms of -log(*p*-value). The blue points correspond to the best SNPs using the grplasso method and the red point corresponds to the best SNP from the single-locus analysis.

Figure 1 shows the results from the bootstrap-penalized regression procedure (left panels) as compared with a standard single-locus analysis (right panels), using windows of 1000 markers around the locations of significant associations detected by Plenge et al. [8]. Analysis of a single region (1000 markers, 50 bootstrap replicates, and a single value of *λ*) took between 10 and 12 hours; this increased significantly with the number of markers (e.g., up to 3 weeks when using 5000-7000 markers). The penalized regression procedure did not appear to offer any great advantage over the single-locus analysis with respect to either detection or localization of the putatively associated polymorphisms. We also examined the value of the estimated penalized regression coefficient at each SNP (for which no bootstrapping was required) when using windows of either 1000, 2000, 5000, or 7000 SNPs (data not shown). Again, no clear advantage over single-locus analysis, with respect to either detection or localization of putative causal variants, was observed.

## Discussion

Penalization approaches are an appealing alternative to standard regression techniques for analysis of large numbers of predictor variables in the context of genome-wide association studies. Use of such techniques is just beginning to emerge: ridge regression [11] has been used for distinguishing between causative and non-causative variants for quantitative phenotypes, and penalized logistic and least angle regression have been used for identifying gene-gene interactions in binary traits [7,12]. A closely-related Bayesian penalized regression procedure [13] has also been suggested for genome-wide and/or fine-mapping studies. Although, theoretically, the simultaneous inclusion of many markers across the genome in a single regression analysis has some appeal (on account of the reduction in residual variance that can be achieved), it is unclear whether one would genuinely expect this to improve upon single-locus analysis with respect to *detection *of disease-associated polymorphisms. A more promising application is the *fine-mapping *problem, in which one is interested in determining from a smaller (although still potentially large) set of strongly correlated predictors in a region, which ones drive the association and are thus potentially causal or lie close to causal variant(s). Simulations suggest that penalized regression may offer some improvement over single-locus methods in this regard [11,13], although interpretation is complicated by difficulties in defining criteria for "true" and "false" detections in this context. In the analyses described here, we did not find the group lasso approach to offer any advantage over single-locus methods with respect to either detection, or localization, of disease-associated polymorphisms. Single-locus analysis provided a clear and localized signal of association, whereas the penalized approach generated a number of somewhat isolated signals, some with unusually small *p*-values, across the regions investigated. Further investigation (data not shown) suggests that use of a higher penalty may produce better results: ideally one might wish to use cross-validation to choose the best value of *λ *from a range of possible values; however, this is likely to be prohibitively time-consuming on a genome-wide scale. Further investigation of alternative penalization algorithms and of methods for choosing penalization parameters and assessing significance is warranted.

## List of abbreviations used

GAW: Genetic Analysis Workshop; WE: Hardy-Weinberg equilibrium; LD: Linkage disequilibrium; NARAC: North American Rheumatoid Arthritis Consortium; RA: Rheumatoid arthritis; SNPs: Single-nucleotide polymorphisms.

## Competing interests

The authors declare that they have no competing interests.

## Authors' contributions

PC participated in the design of the study, carried out the statistical analysis, and helped draft the manuscript. HJC conceived of the study, participated in its design, and drafted the final manuscript. Both authors read and approved the final manuscript.
